# Building paths to success: a multilevel analysis of the effects of an emotional intelligence development program on the academic achievement of future teachers

**DOI:** 10.3389/fpsyg.2024.1377176

**Published:** 2024-03-08

**Authors:** Rosa Poveda-Brotons, Andrea Izquierdo, Natalia Perez-Soto, Teresa Pozo-Rico, Juan-Luis Castejón, Raquel Gilar-Corbi

**Affiliations:** Department of Developmental Psychology and Didactics, University of Alicante, Alicante, Spain

**Keywords:** emotional intelligence, higher education, multilevel analysis, academic achievement, intervention program, pre-service teachers

## Abstract

**Introduction:**

The situation generated by the recent pandemic has had several effects on education, one of them being the necessary but hasty efforts of teachers and students to adapt to the demands of a virtual classroom environment. Thus, it is essential to promote the development of digital competencies in future teachers, enabling them to create effective learning situations in the digital medium. Moreover, the European university curriculum includes a set of specific competencies (specific to each degree) and a series of generic competencies, many of which are related to emotional intelligence. Our work emerges from these specific premises and the more general need to develop emotional skills and learning abilities in virtual environments for future educators.

**Methods:**

The sample comprised 240 students in higher education, pursuing a bachelor’s degree in education at the University of Alicante, Spain (68.3% were female and average age of the participants was 19.43 years SD = 4.127). Using a quasi-experimental design with pretests, posttests, and a control group, we present the effects of an online program aimed at improving the emotional skills and academic achievement of future teachers in higher education. In this study, a 30-h emotional intelligence training program was implemented. Since the student sample was grouped into different classes, we analyzed, using a generalized linear mixed-effects model, whether students who took the program showed a significant improvement in their academic achievement at the end of their studies, compared to those who did not take it.

**Results:**

The findings show a significant improvement in the academic achievement at the end of the bachelor’s degree studies of the students in the experimental group.

**Discussion:**

These results open up a field of possibilities for the implementation of this type of training using virtual environments, enabling interventions to enhance the emotional development of the teaching staff (and, subsequently, in their students), develop their teaching careers adequately, and employ the ideal strategies to address educational programs. Looking ahead, the integration of emotional development programs that incorporate emerging technology into university curricula could enhance the preparation of educators profoundly and create adaptable learning environments for contemporary students.

## 1 Introduction

### 1.1 Emotional intelligence and its assessment

The concept of emotional intelligence (EI) was initially proposed by Salovey and Mayer (1990). A few years later, the aforementioned researchers revisited the concept and introduced a new dimension of EI (understanding emotions), ultimately concluding that the mental processes of the new EI model included the perception, appraisal, and expression of emotions; the emotional facilitation of thought; emotional understanding; and emotional regulation (Mayer and Salovey, 1997).

Since then, there has been considerable controversy over defining EI in numerous studies. Consequently, the scientific community has divided the different models into three major categories: ability models, mixed models, and trait models. Nevertheless, Petrides and Furnham (2001) and Pérez et al. (2005) suggested that these models are often complementary rather than contradictory. Mayer et al. (2000) characterized ability models as those focusing on the interaction between intelligence and emotion, as traditionally defined, and mixed models as those using questionnaires that combine the measurement of personality traits and particular emotional competencies (Goleman, 1996; Bar-On, 2002a). Conversely, Petrides and Furnham (2000) proposed considering a trait model when EI is assessed through self-reports, stating that such measures do not evaluate a person’s ability to perform a task but, rather, assess the extent to which a person reports on their behavioral habits.

Consequently, various instruments exist to assess EI, depending on what aspects are to be evaluated. In this study, two types of measures were primarily employed: trait-based and ability-based EI, each with its advantages and disadvantages. In the case of trait-based EI, assessed through self-reports, a disadvantage is noted, as participants may not be accurate in assessing their emotional competencies (Boyatzis, 2018) or may falsify their responses (Tett et al., 2012). Conversely, this is not an issue with ability-based EI, as participants are asked to select the correct response to a scenario in which they must demonstrate emotional competence. Furthermore, while trait-based EI exhibits better psychometric properties (O’Connor et al., 2019), Miao et al. (2017) suggested that assessments of EI ability exhibit weak predictive power for the anticipated outcomes.

### 1.2 The significance of EI in diverse settings

The advantages of EI have been recorded in different facets of life, with a particular emphasis on professional fields (Stys and Brown, 2004). Currently, society is focusing on promoting this intelligence concept because training in EI results in improvements in both personal and social adjustment across all developmental stages of an individual and their interaction with their environment (Bisquerra and Mateo, 2019). Notably, as asserted by Brackett et al. (2011), EI contributes to overall well-being in personal, family, work, and social domains, leading to an enhancement in satisfaction with oneself and others.

Chiva and Alegre (2008) illustrated a positive correlation between self-reported EI (based on the mixed model) and job satisfaction, implying that individuals with elevated EI levels tend to experience greater job satisfaction. Moreover, a similar study, also using the mixed model, indicated that individuals who are adjudged more emotionally and socially competent demonstrate greater self-control in their professional and personal lives (Sener et al., 2009). In summary, according to the mixed models, EI strengthens an individual’s capacity to address and manage demands effectively in their environment (Moreno-Jimenez et al., 2014) and influences the development of more assertive communication, resulting in a healthier work environment (Froman, 2010).

Some studies have also linked trait EI with various work-related attitudes, such as work effectiveness (O’Boyle et al., 2011), satisfaction, and organizational commitment (Miao et al., 2017). Mavroveli and Sánchez-Ruiz (2011) and Petrides et al. (2016) linked trait EI to a wide array of social and emotional skills in individuals across various age groups, including children and adults.

Regarding ability models, some studies position EI as a predictor of individual well-being, professional success, and the attainment of superior social competencies (Hur et al., 2011; Nelis et al., 2011).

In the field of education, recognizing that the development of EI brings about various benefits for individuals, there is a growing interest in studying its effects and relationships with, among others, stress (Oberle et al., 2020; Schaefer et al., 2020); motivation toward learning (Al-Qadri and Zhao, 2021; Arias et al., 2022); social support from family, teachers, and peer groups (Antonio-Aguirre et al., 2019); student behavior (Durlak et al., 2011); and burnout (Arvidsson et al., 2019; Vicente de Vera and Gabari, 2019; Meredith et al., 2020). Simultaneously, the scientific community is increasingly recognizing the impact of EI on students’ academic achievement across all educational stages (Pulido-Acosta and Herrera-Clavero, 2019; Costa and Faria, 2020; Fontanillas-Moneo et al., 2022). Noteworthy, too, are those studies examining the relationship between EI and teacher effectiveness, asserting a positive correlation between teachers’ well-being and effectiveness in the teaching process (Herman et al., 2017; Triff et al., 2019).

The relationship between the EI of teachers and their efficacy in the classroom, as articulated by Sutton and Wheatley (2003) and Jennings and Greenberg (2009), underscores the pivotal role of emotions in educational instruction and learning procedures. The aforementioned researchers not only highlighted the connection between teachers’ EI and quality education but also documented the fostering of prosocial behavior in students. Meanwhile, Di Fabio and Palazzeschi (2008) explored the connection between EI and self-efficacy in Italian teachers. Their findings suggested that the best explanation for teacher self-efficacy lies in the intrapersonal dimension. Chan (2006, 2008) suggested that improved EI could alleviate burnout and predicted coping strategies among Hong Kong teachers, emphasizing both intrapersonal and interpersonal intelligence. Colomeischi and Colomeischi (2014) underscored the importance of EI, self-efficacy, work mindset, and job satisfaction in developing strategies to enhance teacher training programs.

In an Asian context, Lee and Yin (2011) explored the emotional reactions of educators to changes in the educational system in Chinese secondary education, identifying diverse emotional profiles conducive to effective curriculum management. A subsequent work by Yin and Lee (2012) set out four key guidelines for emotional expression governing Asian teachers: teaching commitment with enthusiasm, concealing unfavorable emotions, regulating negative feelings, and managing emotions effectively to attain teaching objectives.

### 1.3 Positive effects of EI in higher education

There is widespread agreement regarding the significance of cultivating social intelligence and EI (Bar-On, 2000; Domitrovich et al., 2007; Nelis et al., 2009; Palomera et al., 2017). Nevertheless, the nurturing of this competency necessitates active participation from the entire educational community and spans all educational stages, including university education. Owing to the relationship between schools and universities, advocating for a simultaneous transformation of both is imminently logical, with teacher training constituting a key aspect of this process (Ibáñez et al., 2008).

Various institutions often face substantial difficulties in implementing initiatives, as highlighted by Elias et al. (1997) and Zins et al. (2004). The 1999 Joint Declaration of the European Higher Education Area emphasized the importance of students acquiring skills, values, and competencies to establish a cohesive European space for higher education (Ministros Europeos, 1999). The Tuning project in 2003 defined the professional profiles associated with each area of study, emphasizing the acquisition of desirable competencies, including those associated with EI, such as social skills and collaborative teamwork. In current European university curricula, a collection of particular competencies (customized for each degree) and general competencies are incorporated to prepare students for engaging in the specific profession associated with their degree, aligning with industry demands. Nevertheless, research conducted with university students indicates a deficiency in those competencies sought by companies for successful integration into the job market. Employers express a demand for a broader range of competencies than those which graduates typically demonstrate (Agencia Nacional de Evaluación de la Calidad y Acreditación [ANECA], 2007; Pertegal-Felices et al., 2011). The existing research demonstrates that students often lack sufficient capacity to control their emotions, work in teams, manage others, and adapt to continuous change (Pertegal-Felices et al., 2011, 2014). This deficiency is closely tied to decision-making, an aspect that, as Li et al. (2020) suggested, could be fostered through EI.

### 1.4 The significance of emotional intelligence in academic achievement and teacher training

The emotional profile of university students has been explored extensively in studies linking it to burnout syndrome (Extremera and Durán, 2007; Weinstein, 2011) as well as the cultivation of emotional skills demanded by organizations (Molero and Reina-Estévez, 2012; Pertegal-Felices et al., 2014) and academic achievement (Parker et al., 2004; Caruso and Howe, 2015). Researchers such as Barchard (2001), Brackett and Mayer (2003), and Lam and Kirby (2002) have found a moderately strong correlation between EI and academic achievement, using grades as an outcome measure. According to Parker et al. (2004), it is crucial to consider the importance of emotional and social competence in academic achievement. Their work involved measuring academic success and EI in a sample of secondary school students, demonstrating a robust correlation between academic achievement and the various dimensions of EI.

Nevertheless, as highlighted by Zeidner et al. (2002), the existing body of research is inadequate to establish definitively that students with elevated levels of EI in specific dimensions attain superior academic achievement. This paucity of reliable scientific evidence can be ascribed to conflicting or inconclusive findings, often stemming from challenges met in the assessment procedures (Newsome et al., 2000); a lack of awareness regarding available and suitable assessment tools spanning scientific, educational, clinical, and organizational domains (Extremera et al., 2004); methodological disparities inherent in many studies (Parker et al., 2004); and the organizational contexts in general educational centers and systems (Extremera and Durán, 2007). Corcoran et al. (2018) also suggested that, although improvements in academic achievement are demonstrated after a review of various interventions, further studies are required to consider the benefits of EI for students’ academic success.

Acknowledging the significance of EI, it has been increasingly confirmed that EI is an aspect related to an individual’s achievement capacity and can be trained and taught (Gilar-Corbi et al., 2008; Pope et al., 2013). According to Nelis et al. (2009), EI can be improved through training; these researchers found that after 18 h of training, participants improved significantly in the domains of emotional regulation, emotional understanding, and overall emotional competencies.

In light of the perceived need to foster emotional competencies, integrating their development into educational curricula, particularly through teacher training, is considered essential for effective professional growth (Pertegal-Felices et al., 2011; Palomera et al., 2017). Several notable interventions by various researchers include those by Bond and Manser (2009), Oberst et al. (2009), Yilmaz (2009), and Short et al. (2010). Short et al. (2010) implemented a program where coaching specialists guided psychology students on matters related to well-being, yielding lower stress levels in the intervention groups; however, its influence on academic achievement was not investigated. Yilmaz (2009) reduced anger levels effectively in university students through specific anger management training. Other initiatives, such as Shek et al. (2012) “Leader of Tomorrow” program and Pool and Qualter’s (2012) intervention at an English university, successfully enhanced overall EI and emotional self-efficacy among students in the experimental group.

### 1.5 Training in virtual environments

The situation created by the recent pandemic has had various effects on 21st-first-century education, one of which is the effort that teachers and students have had to abruptly adapt to the demands of a virtual classroom environment (Kaden, 2020; Van Nuland et al., 2020; Watermeyer et al., 2021).

Research, including studies by Sánchez and Alemán (2011), and Valtonen et al. (2015), has shown that integrating information and communication technologies (ICT) into the teaching and learning process enhances the quality of education. Nevertheless, the integration of ICT into the educational system presents a substantial professional challenge for teachers, as noted by Drossel and Eickelmann (2017), as teachers need to develop digital competencies that enable them to create effective learning situations in the digital medium (Gilar-Corbi et al., 2018a, 2019b). In this regard, according to Le (2022), teachers must follow the most suitable methodology for the effective design of activities offered to students in a virtual environment.

Furthermore, there is a growing trend in providing online training to equip participants with tools and resources that both practicing teachers and teacher trainees can implement. The goal is to enhance teacher preparation for creating adaptable learning environments for the digital students of today, promoting direct and interactive contact with students (Beltrán et al., 2017), and using ICT for educational purposes. This approach should be addressed at every educational level, including higher education.

This concern has also reached UNESCO, where a Framework of ICT Competency for Teachers has been proposed (United Nations Educational, Scientific, and Cultural Organization, 2019). The objective is to guide teachers in their pre-service and in-service training regarding the use of technologies in formal and informal education. The framework consists of 18 digital competencies that encourage teachers to understand national policy priorities with respect to the crucial role of ICT in education, supporting various aspects of the curriculum, assessment strategies, pedagogy, school and classroom organization, administration, and continuous professional development.

### 1.6 Development of EI training

In accordance with the aforementioned and to confirm the need to include EI in the initial training of teachers due to its connection with greater future professional development, this study aims to analyze the effectiveness of virtual training in developing EI in university students training to be teachers. Specifically, a 30-h EI training program is implemented. This program has already been used in previous research, demonstrating a significant improvement in emotional skills assessed in higher education students from different cultures (Gilar-Corbi et al., 2019b). Various methodologies have been employed for the delivery of the training, including online, in-class, and a combination of an e-learning platform with face-to-face tutoring, showcasing its versatility (Gilar-Corbi et al., 2018b). In addition, the effectiveness of this training has already been assessed among primary school teachers (Pozo-Rico et al., 2020). Furthermore, another study demonstrated that EI, evaluated through mixed measures and ability measures, improved after training conducted with top executives of a private company (Gilar-Corbi et al., 2019a).

In this study, only the online modality has been used. That is, the training has been conducted in a virtual environment, facilitated through forums and activities that promote equal dialog between participants. To effectively deliver the emotional intelligence training program, the methodology used a comprehensive approach by incorporating various instructional strategies. Presentations were structured sessions that systematically introduced and explained key concepts, program objectives, and methodological approaches. These presentations provided participants with foundational knowledge and established a framework for subsequent learning activities. Discussions were interactive sessions designed to promote critical thinking, knowledge exchange, and active engagement among participants. Participants had the opportunity to explore different perspectives, share insights, and collectively deepen their understanding of emotional intelligence principles through facilitated dialog and group interaction. Activities such as self-reflection exercises, case studies, and group tasks were integrated to foster experiential learning and practical application of emotional intelligence skills. These activities encouraged participants to apply theoretical concepts to real-world scenarios, analyze complex situations, and develop effective strategies for emotional regulation and interpersonal communication. Furthermore, immersive learning experiences were created through the use of role-playing exercises. These exercises allowed participants to simulate teaching scenarios and practice applying emotional intelligence principles in authentic contexts. The role-plays facilitated the development of empathy, communication, and decision-making skills, while providing valuable insights into classroom dynamics and educator-student interactions. In summary, the methodology utilized various instructional strategies such as presentations, discussions, activities, and role-playing exercises to create a comprehensive and immersive learning experience. This approach effectively prepares future educators to navigate the complexities of teaching in the digital age while fostering emotional intelligence and enhancing academic achievement.

Furthermore, both the mixed model and the ability model of measures are used to assess the level of EI in the participants in this study. This approach provides a measure of the behavioral tendency at an emotional level and an assessment of the emotional competencies possessed by the individuals (O’Connor et al., 2019).

The present study aims to evaluate whether this program, which has already proven to be effective in improving EI, as demonstrated by the existing research (Gilar-Corbi et al., 2018b, 2019a,b), is also effective in enhancing academic achievement in higher education. Therefore, the hypothesis of this study is as follows:

Participation in the EI improvement program will significantly enhance the academic achievement at the end of the university education of students in the experimental group compared to those in the control group.

## 2 Methods

### 2.1 Participants

The sample consisted of 240 higher education students enrolled in the Primary Education Teacher Bachelor’s Degree at the University of Alicante, Spain. Of the participants, 68.3% were female and 31.7% were male. The average age of the participants was 19.43 years (SD = 4.127). The participants were randomly allocated to either the experimental or control groups. However, it is important to note that the students were already naturally grouped in each class group.

### 2.2 Measures

This study employed a dual perspective to assess EI as a perceived trait and as a cognitive ability.

1. The EQ-i:S (Bar-On, 2002b) is an abbreviated Spanish version of the Emotional Quotient Inventory (Bar-On, 1997) developed by Multi-Health Systems Inc. in Toronto, Canada. Comprising 51 items, the EQ-i:S evaluates five EI factors: intrapersonal intelligence, interpersonal intelligence, adaptation, stress management, and general mood. In addition, it provides an overall EI score using a 5-point Likert scale, with a score of 1 indicating “Very rarely true for me” and 5 indicating “Very often true for me.” For example, an illustrative statement is “In handling situations that arise, I try to think of as many approaches as I can.” Bar-On (2002b) affirmed that the EQ-i:S exhibits satisfactory internal reliability, with alpha coefficients ranging from 0.76 to 0.93.
2. The TMMS-24 (Fernández-Berrocal et al., 2004) is an adapted version of the original Trait Meta-Mood Scale (TMMS) developed by Salovey et al. (1995). This scale, comprising 24 items, assesses three factors: emotional attention, emotional clarity, and emotional regulation, with Cronbach’s alpha values of 0.90, 0.90, and 0.86, respectively. The participants responded on a 5-point Likert scale, where 1 represents “strongly disagree,” 2 is “somewhat disagree,” 3 is “neutral,” 4 is “somewhat agree,” and 5 is “strongly agree.” Example items include statements such as “I can often describe my feelings” (item 10) and “When I am sad, I think about all the pleasures in life” (item 19).
3. The Situational Test of Emotional Understanding (STEU) (MacCann and Roberts, 2008) evaluates the comprehension of one’s own emotions and the emotions of others. Although the original version comprises 42 items, a condensed form with 25 items is used here. The reliability coefficient is α = 0.71. The items present different situations in which the participant must identify the emotion felt by the person in the scenario. An example item is “Xavier completes a difficult task on time and under budget. Xavier is most likely to feel…? (a) Surprise (b) Pride (c) Relief (d) Hope (e) Joy.”
4. The State–Trait Emotion Measure (STEM) (MacCann and Roberts, 2008) assesses emotional management. Although the original version consists of 44 items, a shorter version of 20 items was used here. The reliability coefficient is α = 0.92. The items present a contextualized situation to have the participant choose the most appropriate response to manage the proposed situation effectively at an emotional level. An example item is “Surbhi starts a new job where he does not know anyone and finds that no one is particularly friendly. What action would be the most effective for Surbhi? (a) Have fun with his friends outside of work hours. (b) Concentrate on doing his work well at the new job. (c) Make an effort to talk to people and be friendly himself. (d) Leave the job and find one with a better environment.”

Finally, the academic achievement of the university students was taken into account. For this purpose, the grade of the university’s entrance exam was used in the pretest phase; in the posttest phase, the average grade achieved for all the core subjects at the end of the bachelor’s degree was considered.

### 2.3 Procedure

Initially the study protocol received approval from the University of Alicante Ethics Committee (Ref. UA-2021-12-09_2). Subsequently, a request was sent to the professors teaching the first year of the bachelor’s degree, seeking their authorization for the researchers to have access to their classes and to propose to the students the opportunity to participate in the study. Upon obtaining approval from the professors, the researchers entered the authorized classes, explained the study’s objectives to the students, and requested their voluntary participation. Adherence to ethical guidelines was ensured, and all the participants gave written informed consent following the principles outlined in the Declaration of Helsinki.

It Is important to note that students were grouped In class groups, and these initial groupings were maintained. Therefore, class groups were allocated To either The experimental or control conditions using a random process. In The pretest phase, The participants’ EI Was assessed using The previously described measurement instruments: EQ-i:S, TMMS-24, STEU, and STEM. Additionally, information regarding their previous academic achievement (grade of The university’s entrance exam) Was collected. The EI improvement program Was implemented with The experimental group during 7 weeks of The first year of The bachelor’s degree. In The posttest phase, The participants’ EI Was reassessed. Data On academic achievement (average grade of The core subjects) were obtained 3 years after The intervention when The students Had completed their studies and were provided by the data processing center of The University of Alicante.

### 2.4 Intervention program characteristics

The program lasted 7 weeks, with a 90-min online session each week. The content covered in each session was as follows.

The first session introduced the program’s objectives and methodology. The second session focused on intrapersonal EI and self-perception, emphasizing the identification of one’s own emotions. In the third session, interpersonal EI took precedence, concentrating on recognizing emotions in others. The fourth session addressed adaptability and decision-making, targeting the understanding of the impact of emotions on thoughts, decisions, and behavior at work. The fifth session centered on general mood and self-expression, addressing the effective expression of one’s emotions and mood control. Stress management was the focus of the sixth session, emphasizing the ability to control experienced stress. The seventh and final session aimed to enhance emotional understanding and emotion management, focusing on handling one’s emotions effectively and influencing the emotions of others. For detailed information, please refer to Gilar-Corbi et al. (2018b).

### 2.5 Design and data analysis

A pretest and posttest design with a non-equivalent control group was adopted (Campbell and Stanley, 2015). Given that the participants were grouped in classes, the evaluation of the intervention’s effectiveness on academic achievement was conducted using a generalized linear mixed-effects model adjusted for factors related to EI and previous academic achievement measures and the covariate of gender. The significance level was set at α = 0.05, and the null hypothesis was rejected when *p* ≤ 0.05.

All statistical analyses were conducted using the Statistical Package for the Social Sciences, version 21.0.

## 3 Results

As the participants were grouped by class groups, a multilevel analysis was conducted. To assess the effectiveness of the EI improvement program on academic achievement at the end of the participants’ studies, a generalized linear mixed-effects model was employed. The main effects included belonging to the control or experimental group, gender, previous academic achievement, and different measures of EI at the posttest moment. The random effect was the class group, considering the correlation between students in the same class group. Furthermore, a comparison was made with a linear regression model by removing the random effect. The fit indices for both models indicate no significant differences between the two models. The findings for the generalized linear mixed-effects model are shown in Table 1.

**Table 1:** Fit indices for the linear mixed-effects model and linear regression (target: academic achievement).

Fit index	Linear mixed-effects model	Linear regression	Chi-square difference	Sig.
−2LL	300.703	300.908	0.205	0.65
AICC	304.757	302.926		
BIC	311.544	306.328		

−2LL, −2 log likelihood; AICC, Corrected Akaike Information Criterion; BIC, Bayesian Information Criterion.

The results of the fixed-effects test indicate that the model and all effects are significant, except for gender and the STEU and adaptability variables. Table 2 displays the parameter estimates for the entire model as well as the individual effects. The coefficients illustrate the connection of each parameter in the model to academic achievement. Holding the other parameters constant, we would expect the academic achievement of a student in the experimental group to be 1.127 points higher than a student in the control group. For continuous variables, the coefficient indicates the expected change in academic achievement for a one-unit increase in that variable. For example, in the case of prior academic achievement, holding the other parameters constant, we would expect that for each unit increase in prior academic achievement, the student’s final academic achievement would increase by 0.101 points. Similarly, we would expect that for each unit increase in the scores of the variables attention, clarity, regulation, STEM, intrapersonal intelligence, interpersonal intelligence, stress management, and mood, the student’s final academic achievement would increase by 0.029, 0.025, 0.021, 0.135, 0.021, 0.024, 0.017, and 0.022 points, respectively.

**Table 2:** Fixed coefficients.

Model terms	Coefficient	SE	*t*	*Sig.*	95% CI	
Intersection	−1.81	0.73	−2.485	0.014	−3.262	−0.375
Type of intervention	1.127	0.09	11.34	<0.0001	0.93	1.322
Prior achievement (entrance grade)	0.101	0.02	4.63	<0.0001	0.05	0.14
Gender	−0.04	0.05	−0.72	0.470	−0.14	0.06
Attention	0.02	0.01	3.03	0.003	0.01	0.04
Clarity	0.02	0.01	2.40	0.017	0.004	0.04
Regulation	0.02	0.01	2.41	0.17	0.004	0.03
STEU	0.02	0.03	0.66	0.505	−0.05	0.10
STEM	0.13	0.04	2.91	0.004	0.04	0.22
Intrapersonal	0.02	0.01	2.38	0.018	0.004	0.03
Interpersonal	0.02	0.01	2.23	0.026	0.003	0.04
Stress management	0.01	0.01	2.07	0.039	0.001	0.03
Adaptability	0.01	0.01	1.35	0.17	−0.007	0.03
Mood	0.02	0.009	2.44	0.015	0.004	0.03

Target: academic achievement.

The results of the analysis of parameter estimates for covariance and related statistics for residual and random effects showed a unique residual variance estimate of 0.14 (SE = 0.01, z = 10.53, *p* < 0.001, 95% CI: 0.12 to 0.17). However, variance estimates for the intercept effect were 0.002 and were not statistically significant (SE = 0.006, *z* = 0.35, *p* = 0.72, 95% CI: 0.000 to 0.39), indicating no class group effect in the model.

**Figure 1:**
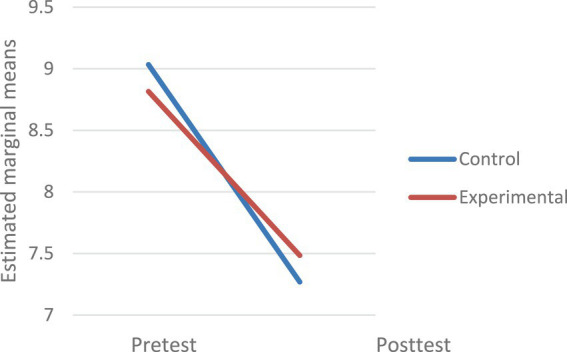
Interaction plot for academic achievement score.

Figure 1 shows the interaction plot, illustrating the direction of differences. Although the control group started with a slightly higher academic achievement before the study (the university entrance grade), the academic achievement of the students in the experimental group is significantly higher at the end of their studies than that of those in the control group In both groups, university entrance grade are higher than the average grade obtained in the bachelor’s degree, which explains their decrease between the pretest and posttest moments, both in the experimental group and the control group. However, in the experimental group, this decrease was significantly lower, with the grades at the end of the bachelor’s degree of the students who participated in the emotional intelligence development program being significantly better.

## 4 Discussion

Several studies underscore the significance of EI in career progression, including those by Cooper (1997), Murga and Ortego (2003), Boyatzis (2006, 2008), Dreyfus (2008), and Koman and Wolff (2008). In the field of education, the research indicates that the effectiveness and quality of teachers are linked to their levels of EI, as demonstrated by Sutton and Wheatley (2003), Di Fabio and Palazzeschi (2008), and Jennings and Greenberg (2009). In addition, these investigations, among others, highlight the various benefits of EI, such as enhancements in prosocial behavior among practicing teachers, job satisfaction, or more effective approaches to enhancing educational programs (Chan, 2008; Colomeischi and Colomeischi, 2014). Furthermore, as noted by Bisquerra et al. (2015), it is advisable to promote the emotional development of teachers first so that said teachers can then enhance the EI of their students.

In the educational setting, it is crucial to highlight that enhancing teachers’ fundamental skill of identifying emotions in others contributes to the cultivation of specific capabilities essential for skillful conflict resolution, particularly in addressing potential disputes among students (Extremera and Fernández-Berrocal, 2004). Furthermore, possessing the ability to perceive emotions serves as a foundational skill for implementing effective emotional regulation strategies and is correlated with the capacity to respond empathetically to others (Mayer et al., 1990). In general, teaching is enhanced whenever overall well-being is promoted among the teaching staff (Liang et al., 2020). Moreover, Huamán et al. (2020) highlighted the connection between the EI of university faculty and the academic satisfaction of students.

Seoane-Pardo and García-Peñalvo (2014) emphasized that teaching has been considered a skill that arises from both experience and certain emotional capabilities. Therefore, it is relevant and necessary for the teacher to possess technical, academic, and, above all, EI competencies. In the current technological environment, technical competencies are required, but the ability to manage emotions and establish meaningful connections with students is positioned as a key factor for excellent performance (Goleman, 1998). Various studies indicate that emotional competencies make for significant differences between ordinary and outstanding workers (Goleman, 1998). In the educational context, these competencies could be the differentiating factor between an ordinary teacher and a highly effective one (Pagano, 2008). Deliberately and systematically incorporating EI into teacher training not only enhances the quality of instruction but also contributes to the broader educational environment, fostering a comprehensive approach to students’ learning and well-being.

Considering all the aforementioned factors, this study introduces a program designed to elevate EI levels and academic achievement among teachers in training. The aim is to equip these teachers with enhanced skills as they embark on their professional journeys as future educators. The findings indicate the feasibility of improving EI among college students and that this improvement has a positive impact on their academic achievement, aligning with studies such as those conducted by Dong et al. (2022) and MacCann et al. (2020). In summary, our findings indicate that EI has the potential to be cultivated in university education and that the higher education setting offers an optimal atmosphere to enhance emotional regulation, thereby bolstering various learning encounters.

Based on the notion that teaching a skill not previously attained is impossible, Weare and Gray (2003) suggested the explicit development of socioemotional skills in institutions offering teacher training. The existing research emphasizes the significance of personal and socioemotional skills in the professional work of teachers, pointing out a challenge for higher education institutions: the commitment to promote EI to enhance the professional development of educators (García-Martínez et al., 2021). Some studies (Sutton and Wheatley, 2003; Jennings, 2011) have highlighted the intimate connection between teachers’ socioemotional skills and their effectiveness in classroom teaching and learning processes. The literature suggests that teachers can successfully navigate the challenges posed by the educational context when equipped with adequate levels of EI (Berkovich and Eyal, 2015; Ju et al., 2015; Wurf and Croft-Piggin, 2015; Yin, 2015).

Moreover, online education offers a dynamic platform that transcends geographical barriers and time constraints, providing a flexible learning environment suitable for diverse student populations. The digital landscape facilitates accessibility, enabling educators to reach a broader audience with tailored programs. In the context of EI training, online platforms offer interactive tools, simulations, and multimedia resources that engage learners actively. In addition, the asynchronous nature of online learning allows individuals to pace their progress, fostering a self-directed approach to skill development. The integration of emerging technologies in EI programs not only enriches content delivery but also cultivates digital literacy skills among future educators. Moreover, online platforms enable collaborative learning experiences, encouraging the exchange of perspectives among students from varied backgrounds, thereby enriching their EI.

In line with this rationale, recent research, exemplified by the study conducted by Pozo-Rico and Sandoval (2020), underscores the effectiveness of online e-learning tools in nurturing EI among prospective educators. This study notably reported a marked increase in academic achievement and considerably higher levels of teacher satisfaction in the online e-learning group compared to traditional instructional methods. The flexible, accessible nature of online platforms not only facilitates comprehensive engagement but also fosters the development of EI competencies essential for educators’ holistic growth. Similarly, Gilar-Corbi et al. (2018b) underscored the substantial success and adaptability of the online learning component in a comprehensive EI program. Furthermore, the aforementioned researchers accentuated the significance of attaining not only technical prowess but also essential competencies such as effective communication, teamwork, adept time management, and the crucial ability to regulate emotions. In a similar vein, numerous studies (Greenhow et al., 2022; Salas-Pilco et al., 2022; Tarchi et al., 2022) have noted the advantages and feasibility of online learning, emphasizing its crucial role in navigating the shifting terrain of higher education. Such investigations underscore the importance of embracing innovative methodologies that transform e-learning spaces into catalysts for student engagement and care across behavioral, cognitive, and affective dimensions.

In conclusion, enhancing the emotional skills of higher education students has a positive impact on their learning and academic achievement. In addition, it is crucial to focus on the emotional competencies of future educators for the evolution and improvement of teaching practices. Going forward, the integration of emotional development programs that incorporate emerging technology into university curricula could profoundly enhance educators’ preparation and create adaptable learning environments for present-day digital students. This integration would not only strengthen educators’ preparation and academic achievement but also positively impact learning quality, student well-being, and the educational system’s ability to adapt to the changing demands of contemporary society.

Moreover, as we navigate an ever-evolving educational landscape, recognizing EI as a cornerstone for both student and educator success becomes an imperative. Extending beyond traditional academic prowess, fostering emotional skills among educators and learners is instrumental in cultivating an inclusive and supportive learning ecosystem. Given the rapid advancements in technology, a forward-looking approach involves harnessing emerging tools and platforms to fortify emotional development programs in university curricula. By integrating innovative technological solutions alongside EI initiatives, educators can not only empower students with vital emotional competencies but also equip themselves with the adaptive skills required to navigate a digitally driven educational sphere.

Nevertheless, it is important to acknowledge that the program studied herein was exclusively implemented in a sample of participants studying for a specific bachelor’s degree. Given the program’s potential applicability across various university degrees and to address this limitation, a critical step involves expanding its implementation to encompass a diverse range of university degrees, thereby transcending disciplinary boundaries. This expansion is crucial to enable a more comprehensive evaluation of its efficacy and adaptability in varying academic contexts. Collaborative initiatives between faculty members from distinct disciplines play a pivotal role, as they facilitate the customization and fine-tuning of specific modules to meet the specific requirements and unique contexts of each field. Furthermore, conducting comparative analyses across diverse academic disciplines will yield invaluable insights into the nuanced ways in which interventions targeting EI affect learning across varied domains in higher education. Consequently, the forward trajectory of this study involves embracing this inclusive approach, allowing the program to undergo rigorous assessments that ensure its relevance and effectiveness in nurturing EI skills tailored to suit the multifaceted landscapes of higher education.

## Data availability statement

The raw data supporting the conclusions of this article will be made available by the authors, without undue reservation.

## Ethics statement

The studies involving humans were approved by the University of Alicante Ethics Committee (Ref. UA-2021-12-09_2). The studies were conducted in accordance with the local legislation and institutional requirements. The participants provided their written informed consent to participate in this study.

## Author contributions

RP-B: Investigation, Writing – original draft, Data curation. AI: Conceptualization, Writing – original draft, Data curation. NP-S: Investigation, Writing – original draft. TP-R: Investigation, Methodology, Writing – original draft. J-LC: Conceptualization, Methodology, Supervision, Writing – original draft. RG-C: Conceptualization, Funding acquisition, Investigation, Methodology, Writing – original draft, Writing – review & editing, Formal analysis.
